# Recombination of N Atoms in a Manifold of Electronic States Simulated by Time-Reversed Nonadiabatic Photodissociation Dynamics of N_2_

**DOI:** 10.1021/acs.jpclett.3c00666

**Published:** 2023-05-11

**Authors:** Natalia Gelfand, Francoise Remacle, Raphael D. Levine

**Affiliations:** † The Fritz Haber Center for Molecular Dynamics, Institute of Chemistry, The Hebrew University of Jerusalem, Jerusalem 91904, Israel; ‡ Theoretical Physical Chemistry, UR MolSys B6c, University of Liège, B4000 Liège, Belgium; § Department of Molecular and Medical Pharmacology, David Geffen School of Medicine, University of California, Los Angeles, California 90095, United States; ∥ Department of Chemistry and Biochemistry, University of California, Los Angeles, California 90095, United States

## Abstract

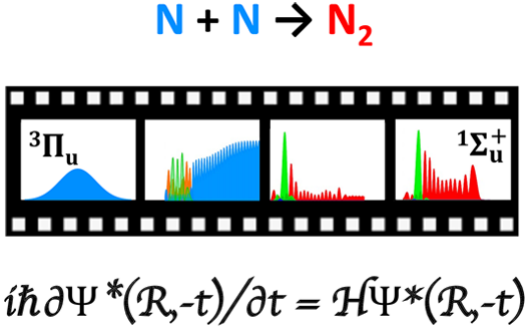

Following a single photon VUV absorption, the N_2_ molecule dissociates into distinct channels leading to N atoms of different reactivities. The optically accessible singlets are bound, and dissociation occurs through spin–orbit induced transfer to the triplets. There is a forest of coupled electronic states, and we here aim to trace a path along the nonadiabatic couplings toward a particular exit channel. To achieve this, we apply a time-reversed quantum dynamical approach that corresponds to a dissociation running back. It begins with an atom–atom relative motion in a particular product channel. Starting with a Gaussian wave packet at the dissociation region of N_2_ and propagating it backward in time, one can see the population transferring among the triplets due to a strong nonadiabatic interaction between these states. Simultaneously, the optically active singlets get populated because of spin–orbit coupling to the triplets. Thus, backward propagation traces the nonradiative association of nitrogen atoms.

Even for the simpler diatomic molecules, there is a maze of higher energy excited electronic states.^1^ High resolution spectroscopy has made major steps in understanding the structure of the stationary states.^2,3^ But the dynamics of these states is still a subject of active research; see, for example, the literature^4−8^ and, of course, the very elegant attosecond studies of H_2_ and HD and their ions.^9,10^ There have been corresponding developments in theoretical and computational dynamics.^11−14^ Two dimensional and higher order spectroscopy has also made significant contributions.^15,16^ Laser methods^17−19^ have enabled a detailed analysis of what are the exit channels. Toward these developments, we discuss a complementary computational approach to photodissociation dynamics. It is here applied to molecular nitrogen, N_2_, in the VUV region,^20−23^ for which very detailed experimental studies of the branching into different electronic exit channels are available.^24−26^

The singlet ^1^Σ_*u*_^+^ states of N_2_ that are optically accessible by one photon VUV spectroscopy are energetically not able to dissociate at these energies. Rather, the dissociating states are of higher multiplicity, primarily triplets and quintets. They are accessed by spin–orbit coupling from the singlet states.^27−29^ The states of given symmetry and multiplicity are mixed by strong nonadiabatic terms.^30^ The considerable spatial and state selectivity of the spin–orbit coupling; see, for example refs (2 and 31), is displayed for N_2_ in ref (32) and here in Scheme 1.

**Scheme 1:**
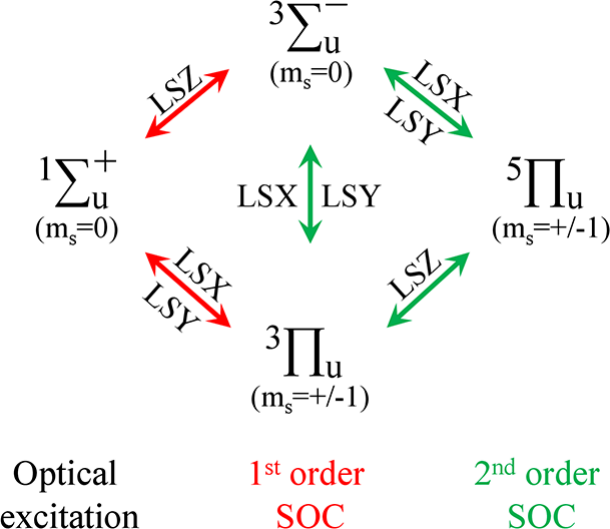
Spin–Orbit Couplings between the Electronic States of N_2_ Included in the Hamiltonian Singlet ^1^Σ_*u*_^+^ states are coupled to triplet ^3^Σ_*u*_^–^ and ^3^Π_*u*_ (1st order coupling), the triplets ^3^Σ_*u*_^–^ and ^3^Π_*u*_ are coupled to one another and to quintet ^5^Π_*u*_ (2nd order coupling). The magnetic quantum number for each electronic state, *m*_*S*_, is indicated in parentheses. The different columns are for different spin multiplicities.

The experiments^24−26^ that resolve the dissociation into final channels clearly show that the branching fraction into each channel depends on the energy and/or the mass in a distinctly nonmonotonic fashion. Furthermore, it makes a difference if the initially optically excited state has a Σ or a Π character and, for each symmetry, if it has primarily a valence excited or a Rydberg character. The stationary singlet states are often a nonadiabatic mixture of these two bonding characteristics,^20,30^ and typically one is more dominant.

To trace the exit dynamics we here perform a time-reversed propagation of the dissociating N_2_ molecule. Starting from a state localized on a particular exit potential and integrating it backward in time. This highlights the role of the doorways to the individual exit channels. The essential technical implementation is as follows. Let ψ(*R*, *t*) be a wave function that is propagated forward in time: *i*ℏ∂ψ(*R*, *t*)/*∂t* = *Hψ*(*R*, *t*). Next, take the complex conjugate of the Schrödinger equation *i*ℏ∂ψ*(*R*, *t*)/∂(−*t*) = Hψ*(*R*, *t*) where the Hamiltonian is Hermitian. Changing *t* to *–t* shows that ψ*(*R*, −*t*) is a wave function propagating backward in time,^33^
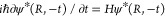
1

Explicitly, for a diatomic molecule already fairly well separated into atoms such that the potential energy is more or less flat, we represent the initial state for the propagation reversed in time as a Gaussian wave packet along the interatomic distance *R* of width *σ*_*R*_. Following the notation of Heller,^34,35^ we have a parameter α that specifies the width, Im(α) = 1/4*σ*_*R*_, of the coherent distribution of momenta about its mean. After taking the complex conjugate of the Gaussian wave function
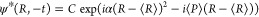
2

We see that the sign of the mean momentum ⟨*P*⟩ has been reversed. This is closely analogous to what one would do to propagate backward in time in a computation using classical dynamics. The three time-independent parameters in the Gaussian wave function, ⟨*P*⟩, ⟨*R*⟩ and α, determine the mean energy of the initial state for the backward propagation and its coherent width as follows. ⟨*P*⟩^2^/2μ is the exit kinetic energy, the total energy minus the threshold energy of that exit channel, and μ is the reduced mass. The wider is the Gaussian in *R*, the narrower is its distribution in momentum about the mean. The specific experiments we aim to simulate resolve different vibrational singlet states so their coherent width in energy needs to be far less than a vibrational spacings in the singlets. The Gaussian begins its journey on the flat part of the exit potential, so we need that ⟨*R*⟩, the initial mean displacement of the Gaussian, is chosen to be significantly larger than the width *σ*_*R*_. In the following we apply these ideas to the nonradiative recombination of a pair of N atoms.

The VUV photodissociation of N_2_ is indirect and proceeds through the triplet and quintet electronic states.^3,24,27,36−38^ We here consider three optically accessible bound singlet ^1^Σ_*u*_^+^ and six dissociative states: one ^3^Σ_*u*_^–^, four ^3^Π_*u*_ and one ^5^Π_*u*_ electronic states (Figure 1A). This set of states was defined based on our recent computational simulation^32^ of the experimentally measured branching fractions into N(^4^S_3/2_) + N(^2^D_*J*_) and N(^4^S_3/2_) + N(^2^P_*J*_) product channels shown with the blue and orange arrows in Figure 1A, respectively. The triplet ^3^Π_*u*_ states have been known to be the primary channels for the photodissociation of the N_2_ molecule.^27,36,37^ A special case is at an excitation energy of 110,144 cm^–1^ where the dissociation of the triplet ^3^Π_*u*_ states is particularly slow and the contribution of the 1^3^Σ_*u*_^–^ and 2^5^Π_*u*_ electronic states is found to be dominant.^32^

**Figure 1:**
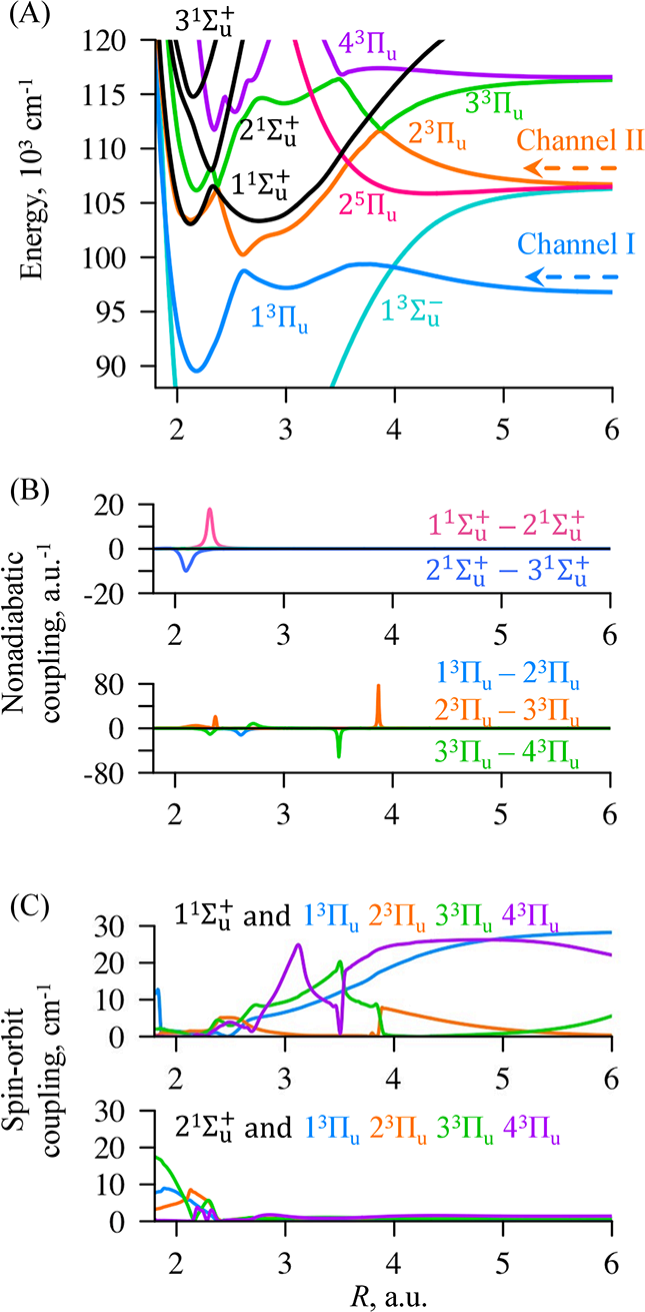
(A) Potential energy curves of the optically accessible singlet ^1^Σ_*u*_^+^ (in black), one ^3^Σ_*u*_^–^, four ^3^Π_*u*_, and one ^5^Π_*u*_ adiabatic electronic states of N_2_ as functions of the internuclear distance *R*. Different electronic states are color coded as shown in the figure. The computed potentials are shifted in energy by 850 cm^–1^ to be in agreement with high resolution spectra as reported in.^20^ The arrows indicate two dissociation channels to N(^4^S_3/2_) + N(^2^D_*J*_) (channel I, blue) and N(^4^S_3/2_) + N(^2^P_*J*_) (channel II, orange). (B) Nonadiabatic coupling terms among singlet ^1^Σ_*u*_^+^ states (top panel in B) and triplet ^3^Π_*u*_ states (bottom panel in B). (C) Spin–orbit coupling terms between triplet ^3^Π_*u*_ states and 1^1^Σ_*u*_^+^ states (top panel in C) and 2^1^Σ_*u*_^+^ states (bottom panel in C).

Each triplet ^3^Π_*u*_ state is degenerate because of its spin multiplicity and its 2-fold spatial degeneracy due to its cylindrical symmetry about the molecular axis. Of the three degenerate spin states only two, *m*_*S*_ = +1 and *m*_*S*_ = −1, can be relevant here because the spin–orbit coupling from the singlet ^1^Σ_*u*_^+^ states does not access the state of zero magnetic quantum number. Similar comments apply to the quintet ^5^Π_*u*_ state. Scheme 1 shows the spin–orbit coupling pattern for the states included in our basis for the backward propagation. In terms of orders of perturbation theory, the singlet states that are optically connected to the ground state are shown as coupled to first order by the spin–orbit terms to triplet states. In second order, the triplet states are spin–orbit coupled among themselves and to the quintet state.

Technically, the computations backward in time start from a Gaussian wave packet localized in the exit channel region on a grid of N–N internuclear distance *R*. We mainly examine time-reversed propagation starting from two different electronic states, 1^3^Π_*u*_ or 2^3^Π_*u*_. For each state of a given total energy, we here take a mixed (meaning not coherent) state where the four degenerate states have each the same amplitude of . We illustrate the time evolution in the population distribution among the coupled triplet and singlet states in Figure 2.

**Figure 2:**
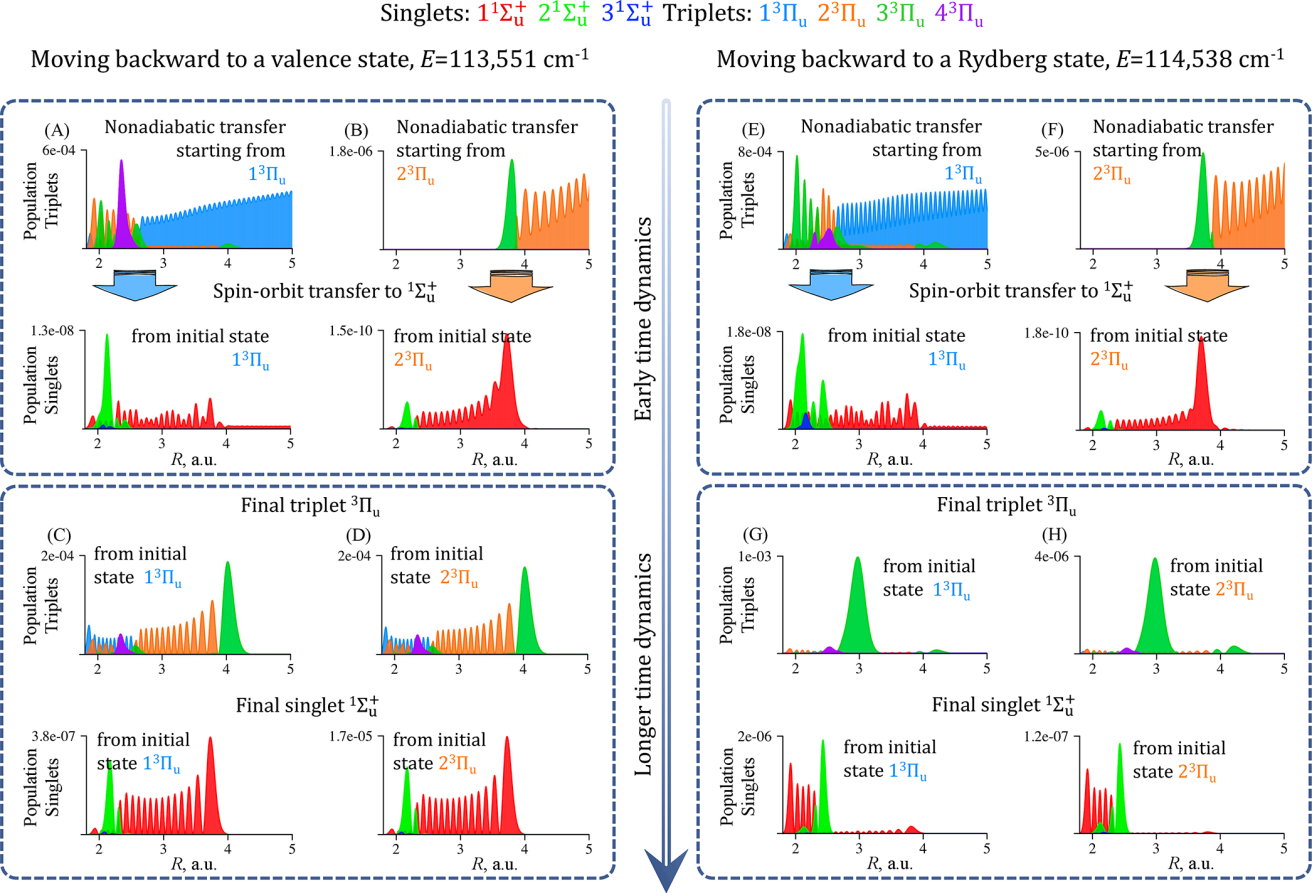
Backward propagation all the way to the singlets. (A, B) Population of triplet ^3^Π_*u*_ states along the grid at early time (*t* = 200 fs) after an initial 1^3^Π_*u*_ (A, top row) or, independently, a2^3^Π_*u*_ (B, top row) state of the same total energy (*E* = 113,551 cm^–1^) and the same width in energy (*σ*_*E*_ = 90 cm^–1^) start moving back to the region of interactions. Singlet ^1^Σ_*u*_^+^ states (A and B, bottom rows) become populated due to slow spin–orbit induced transfer from the triplets. (C, D) Population redistribution of triplet ^3^Π_*u*_ (top rows) and ^1^Σ_*u*_^+^ singlet states (bottom rows) during time-reversed propagation at longer time (*t* = 1000 fs) evolve to a very similar triplet and singlet components. Panels E–H show a corresponding example for the total energy of 114,538 cm^–1^.

Parts A and B (left top panel) of Figure 2 show the early time dynamics when the backward moving initial triplet state reaches the region of interactions, i.e., when the internuclear distance is less than 5 au. At this time the tail of the initial Gaussian wave packet passes the potential energy barriers and transfers population to other ^3^Π_*u*_ triplet electronic states which are coupled by strong nonadiabatic interaction (Figure 1B). The singlet and triplet states are coupled by weak spin–orbit terms (Figure 1C) and therefore the singlet ^1^Σ_*u*_^+^ states are populated from the triplet ^3^Π_*u*_ during the time-reversed propagation. This can be seen from the localization of the population in these states along *R* in Figure 2, parts A and B (computed for 200 fs), and in movies of the dynamics that are included in the Supporting Information.

Propagating an initial triplet state that is narrow in energy makes it possible to access singlet states that correspond to a particular vibrational level of the adiabatic singlet potential. The backward time evolution of the two initial wave packets is driven by sequence of nonadiabatic interactions between the triplets and so are not identical at the early times, compare parts A and B of Figure 2. However, at longer times both initial triplet electronic states, 1^3^Π_*u*_ and 2^3^Π_*u*_ of the same total energy lead back to the singlet and triplet states of a similar character as shown in parts C and D of Figure 2. The final distribution along *R* is similar to the eigenstate of similar energy; see Figure S1 of the Supporting Information. The time evolution of a Rydberg state is shown in Figure 2E–H: the initial redistribution of population is similar to the one shown in Figure 2, parts A and B. Also for this incoming state the distribution at longer time is similar to the eigenstate of the same energy; see Figure S2 of the Supporting Information.

By comparing the localization in the singlet state in Figure 2, parts C and D, one can notice that there is about 50 times more population in the singlet state for the 2^3^Π_*u*_ initial state then for the 1^3^Π_*u*_ one. The two exit channels correspond to N atoms in different electronic states. So, the different yield in N atom recombination is consistent with the expectation of different reactivity for different N atomic states. Therefore, the time-reversed dynamics performed at a particular total energy of the molecule shows the pathway of the electronic system to each channel and the most probable channel for dissociation. More on this in the discussion of Figure 3, below.

**Figure 3:**
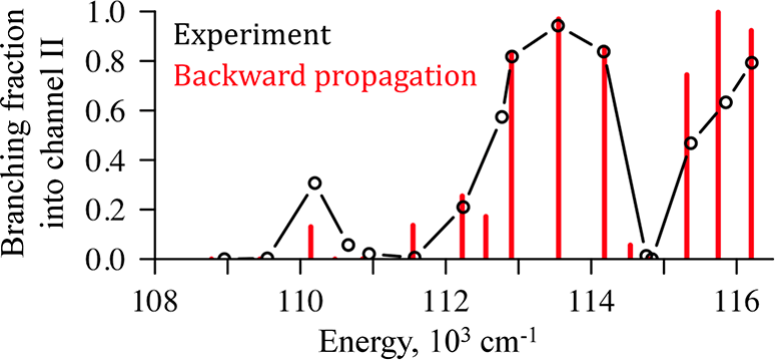
Branching fraction into N(^4^S_3/2_) + N(^2^P_*J*_) (channel II) computed with backward propagation (red sticks) versus experimental data of Song et al.^25^ (black open circles). The dynamics at the energies of 113,551 and 114,538 cm^–1^ that have a rather different branching ratio are shown in detail in Figure 2.

The experiments of Walter et al.^24^ and of Song et al.^25^ showed the nonmonotonic energy dependence of the photodissociation branching ratios of the N_2_ molecule. This experimental result is a laser-induced dynamics at a well-defined wavelength starting from the ground electronic state. Our backward dynamical computation was used to account for the branching as due to the relative reactivity of states of different channels as shown in Figure 3. To quantitatively determine the nonradiative recombination efficiency, we examined the time profile of the population in the *singlet* states as produced by the backward propagation from states in either channels I or II. The evolution backward in time shows an early induction period where population is exchanged followed by a longer time stable population of the singlet states. This can be seen in detail in the movies that are part of the Supporting Information. A particular example is shown in Figure 2, panels C, D, G, and H. We use this distribution to determine the magnitude of the singlet state produced by the recombination. Thereby we determine the branching ratio through the backward computation, where Figure 3 shows a comparison with experiment.

In the energy range from 108,000 to 116,000 cm^–1^ both dissociation channels I and II are open. The first peak into the channel II is at 110,144 cm^–1^ and it is related to a very long predissociation lifetime of the singlet state^24,32^ which allows the triplet 1^3^Σ_*u*_^–^ and quintet 2^5^Π_*u*_ states to contribute to the photodissociation. According to selection rules,^2^^1^Σ_*u*_^+^ and ^3^Σ_*u*_^–^ are coupled by the LSZ spin–orbit term; see Scheme 1. However, the population transfer between ^3^Σ_*u*_^–^ and ^1^Σ_*u*_^+^ is less efficient than that between ^3^Π_*u*_ and ^1^Σ_*u*_^+^. Only when the lifetime of ^1^Σ_*u*_^+^ is rather long, a few hundred nanoseconds, the accumulation due to transfer from ^3^Σ_*u*_^–^ to ^1^Σ_*u*_^+^ can be noticeable.

A significant rise of dissociation into channel II occurs between 112,000 and 114,500 cm^–1^ which is between two avoiding crossing regions: the lower is between 2^3^Π_*u*_ and 3^3^Π_*u*_ at 4 au and the higher one is between 3^3^Π_*u*_ and 4^3^Π_*u*_ at 3.5 au (Figure 1A). Here the singlets become resonant with the repulsive triplet potential formed by 2^3^Π_*u*_, 3^3^Π_*u*_, and 4^3^Π_*u*_ states (Figure 1A). An example of population distribution can be seen in Figure 2A–D.

The shape of potential energy curves of ^3^Π_*u*_ states guides the wave packet. A drastic change in the branching fractions at 114,500 cm^–1^ is induced by trapping of the triplets’ population in the shallow well at 3 au (see the potential of 3^3^Π_*u*_ in Figure 1A). Figure 2E–2H shows both singlets and triplets populated at this energy during backward dynamics. Higher in energy, after 115,000 cm^–1^, the singlets are in resonance with the outgoing states of the repulsive triplet potential.

To summarize, we presented a complementary computational approach that proceeds backward in time from the exit region of the products of the photodissociation into the interaction region. In the backward direction, we start from a high energy electronically excited state so that the energy acquisition in the forward direction by optical excitation from the ground state need not be simulated. The reverse motion at a sharp energy begins with a broad Gaussian wave packet, and its propagation requires only a relatively short time interval to populate all the way back to the singlets. The Supporting Information provides access to movies showing the time evolution of the density of the vibrational state in two different exit channels that are also shown for two time points in Figure 2. Experiments and also our simulations forward in time^32^ demonstrate that the branching fraction into the product channels varies nonmonotonically with the total energy. Figure 3 proves that this is equally the result when we integrate backward. Last, the relative efficiency of nonradiative recombination of N atoms in different electronic states at the same total energy is closely matched by the branching ratios for the photodissociation.

The delayed unimolecular dissociation of electronically excited N_2_ means a longer than simply expected duration for the recombination of two N atoms. It will be a “sticky” collision, and thereby, such mechanisms as a third body induced recombination can be more facile. Computationally our approach allows a more efficient route to exploring the dynamics in the exit region.

## Quantum Dynamical Simulations

We use the time-dependent Schrödinger equation to compute the quantum dynamics for 9 excited electronic states of definite multiplicity. The triplet and quintet states are degenerate because of multiplicity or spatial symmetry. The equation of motion for the amplitudes *C*_*n*, j_^*^ = Ψ_n_^*^(*R*_*j*_) at a given electronic state *n* and grid point *j* are the solutions of
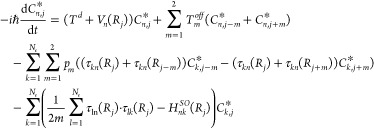
3

Here *T*^*d*^ and *T*^*off*^ are diagonal and off-diagonal kinetic energy terms, respectively, and *V*_*n*_(*R*) denotes the potential energy of an electronic state. The nonadiabatic couplings *τ*_*kn*_(*R*) between electronic states *n* and *k* are scaled by the finite difference momentum terms, *p*_*m*_. We use the five-point finite difference method^39^ to approximate the momentum and kinetic energy terms for the wave function defined on the grid. The total length of the internuclear grid is 67 au with spacing between grid points is 5 × 10^–3^ au. The time step in the propagation is 10^–4^ fs.

The initial state is a Gaussian specified in eq 1, where ⟨*R*⟩ is 37 au with a width of 4.0 au, which is equivalent to *σ*_*E*_ of 90 cm^–1^.

## Supporting Information Available

Comparison between states obtained from diagonalization and dynamics for two examples, total energy of 113,551 and 114,538 cm^–1^. (PDF)
Movie for time-reversed propagation at total energy of 113,551 cm^–1^ using two initial states, 1^3^Π_*u*_ and 2^3^Π_*u*_ (MP4)
Movie for time-reversed propagation at total energy of 114,538 cm^–1^ using (MP4)
