# Supplementary material for: Recombination of N Atoms in a Manifold of Electronic States Simulated by Time-Reversed Nonadiabatic Photodissociation Dynamics of N2
Source: J Phys Chem Lett. 2023 May 11;14(19):4625–30. doi: 10.1021/acs.jpclett.3c00666 (PMC10201567; doi:10.1021/acs.jpclett.3c00666)
Supplement: Supplementary file 1 — jz3c00666_si_001.pdf [file jz3c00666_si_001.pdf]

SUPPORTING INFORMATION

Recombination of N Atoms in a Manifold of Electronic States Simulated by Time-Reversed Nonadiabatic Photodissociation Dynamics of N_2

Natalia Gelfand,[1,] Francoise Remacle,[1,2] and Raphael D. Levine[1,3,4]*

[1] The Fritz Haber Center for Molecular Dynamics, Institute of Chemistry, The Hebrew University of Jerusalem, Jerusalem 91904, Israel

[2] Theoretical Physical Chemistry, UR MolSys B6c, University of Liège, B4000 Liège, Belgium

[3] Department of Molecular and Medical Pharmacology, David Geffen School of Medicine, University of California, Los Angeles, CA 90095, USA

[4] Department of Chemistry and Biochemistry, University of California, Los Angeles, CA 90095, USA

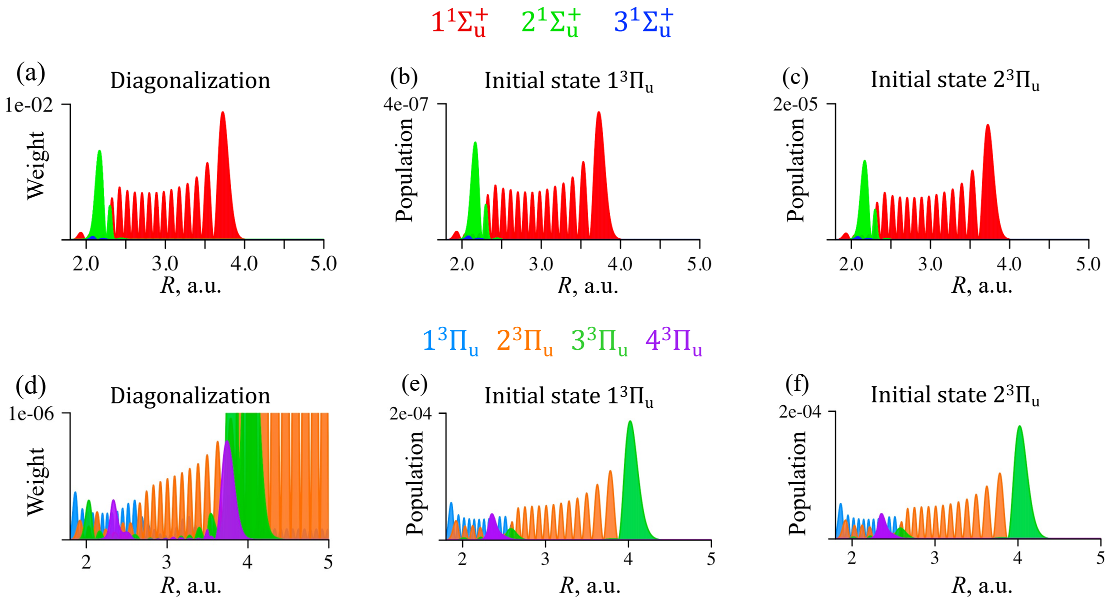

Figure S1. Comparison between singlet (a) and triplet (d) eigenstate from the diagonalization (a, d) for ω = 113,551 cm^{-1} and the singlet and triplet states resulted from the time-reversed dynamics started with initial states $1^3\Pi_u$ (b, e) and $2^3\Pi_u$ (c, f). The population at 1 ps is shown.

*natalia.gelfand@mail.huji.ac.il

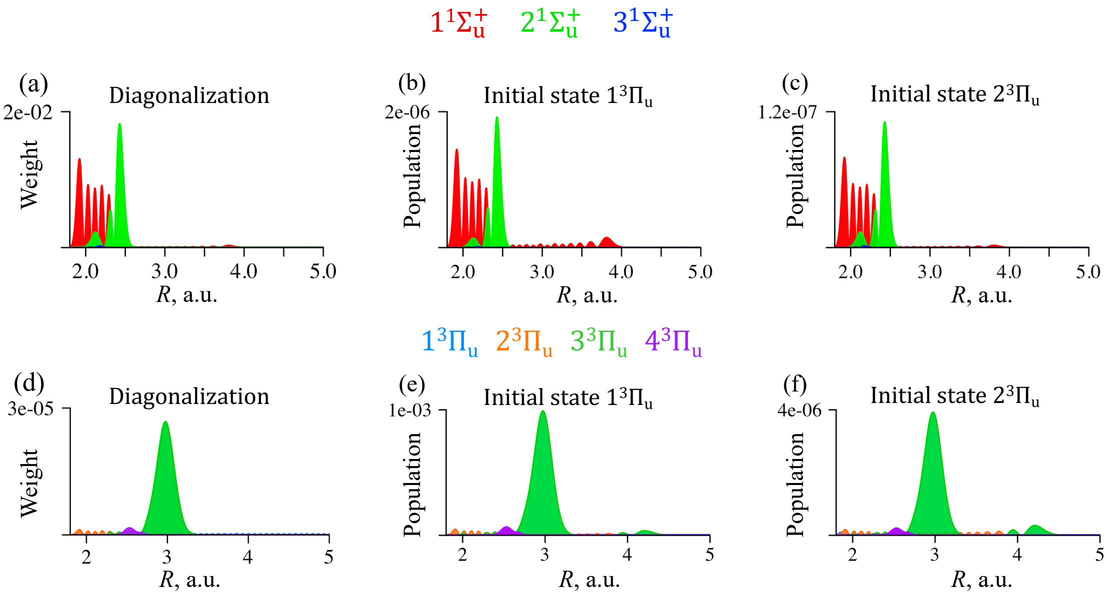

Figure S2. Comparison between singlet (a) and triplet (d) eigenstate from the diagonalization (a, d) for ω = 114,538 cm^{-1} and the singlet and triplet states resulted from the time-reversed dynamics started with initial states $1^3\Pi_u$ (b, e) and $2^3\Pi_u$ (c, f). The population at 1 ps is shown.
